# Value of Exercise Tolerance Testing in Evaluation of Diabetic Patients Presented With Atypical Chest Discomfort

**DOI:** 10.5812/ijem.4284

**Published:** 2012-12-21

**Authors:** Mohammad Esmail Gheydari, Mohsen Jamali, Farhad Hajsheikholeslami, Shahrooz Yazdani, Mina Jamali

**Affiliations:** 1 Cardiology Department, Taleghani Hospital, Shahid Beheshti University of Medical Sciences, Tehran, IR Iran; 2 Research Institute for Endocrine Sciences, Shahid Beheshti University of Medical Sciences, Tehran, IR Iran; 3 Tehran University of Medical Sciences, Tehran, IR Iran

**Keywords:** Exercise Test, Chest Pain, Diabetes, Coronary Angiography, Ischemia

## Abstract

**Background:**

Coronary artery disease is the single most important cause of mortality and morbidity in diabetic patients. Electrocardiographic stress test is a non-invasive modality to screen significant coronary involvement in minimally symptomatic diabetics.

**Objectives:**

We investigated the Positive Predictive Value (PPV) of this test in comparison with coronary angiography.

**Materials and Methods:**

130 diabetic patients with atypical chest discomfort were studied and tested using Exercise Tolerance Test (ETT) among which 100 cases showed positive results that further were studied invasively by selective coronary angiography.

**Results:**

The positive predictive value of ETT for diagnosis of Coronary Artery Disease (CAD)among diabetic patients presented with atypical chest discomfort was 77%.

**Conclusion:**

We conclude that electrocardiographic stress test is a valuable inexpensive non-invasive screening test in diabetic patients with atypical chest discomfort.

## 1. Background 

The perceivable link between the cardiovascular disease (CVD) and diabetes mellitus (DM) had been known for many years. Micro- and macro-vascular complications are accountable in the majority of morbidities and mortalities reported in diabetic patients. Incidence of coronary artery disease (CAD) in diabetics is four times higher compared to the age-adjusted general population (1). Premature initiation and rapid progression of atherosclerosis combined with early commencement of ischemic heart disease (IHD) in association with diffuse involvement of the coronary arteries are common findings in diabetics. As described in earlier studies, CAD, including myocardial infarction (MI), is the leading cause of death in young diabetics (2). Cardiovascular diseases (CVD) are irrefutably responsible for 65% of deaths in diabetics, the majority of whom suffering from type 2 DM (3). In Framingham study it was demonstrated that diabetic individuals exhibited a two to five fold increased risk of developing angina, MI, and congestive heart failure (4). In individuals younger than 45 years, the risk of CVD surges to more than 11 fold compared to general population (5). Mortality statistics after myocardial infarction or even after revascularization procedures are regrettably increased in the setting of preexisting diabetes (6, 7). In most diabetic patients, diagnosis is delayed due to chiefly obscured and atypical symptoms meaning that they don’t meet the usual clinical diagnostic criteria for myocardial ischemia (8). Diabetic patients suffer from more extensive CAD and hence higher incidence of multi vessel CAD than in non-diabetic subjects (9). In DM, early endothelial damage occurs alongside with hyperglycemia, hypertension, dyslipidemia, and hyperinsulinemia (10). Silent ischemia is defined as transient ischemia without typical recognized associated symptoms, especially chest discomfort. Patients with DM experience silent myocardial ischemia more commonly than general population.

As mentioned before, diabetics may suffer from more extensive CAD at an earlier age and silent ischemia contributes to increased adverse outcome. Consequently, detection of CAD before its associated morbidity and mortality is reasonable and may be lifesaving.

Statistics shows that the prevalence of CAD in diabetic patients is on escalation. Screening and detection of high risk coronary lesion with a non-invasive test is an attractive goal. Screening tests should have acceptable sensitivity, specificity, positive predictive value (PPV) and negative predictive value (NPV) to rule in or out significant coronary involvement. Non-invasive tests in addition to detection of CAD should signify the importance of detected lesions. Non-invasive test results should lead us to appropriate Revascularization modality for the detected CAD. Well-known non-invasive tests in detection of IHD include exercise tolerance test (ETT), myocardial perfusion imaging, and stress echocardiography. Each of the above mentioned tests has its own merits in terms of sensitivity and specificity but all are acceptable. ETT is a non-invasive, relatively inexpensive test, and commonly performed in clinical practices. It provides substantial diagnostic and prognostic information. Exercise capacity is one of many important prognostic factors revealed during the test (11, 12).

## 2. Objectives

In this study, we proposed to use the ETT as a screening test for CAD in diabetic patients in one of the teaching university hospitals in Tehran and calculate the positive predictive value of ETT. We also studied the relationship between CAD in diabetic patients with some associated characteristics like hypertension, dyslipidemia, weight, and sex.

## 3. Patients and Methods

The study population consisted of diabetic patients with atypical symptoms of CAD who were referred to out-patient cardiology clinic of Taleghani General Hospital Tehran Iran from January 2010 to January 2011. Diabetes was defined as fasting plasma glucose values ≥ 126 mg/dL, random blood glucose value > 200 mg/dL confirmed at two occasions, history of diagnosed diabetes, or current use of oral hypoglycemic agents or insulin. Diabetic patients with typical symptoms of CAD were excluded; other exclusion criteria consisted of abnormal base line electrocardiogram (ECG), and orthopedic, pulmonary, neurological, and peripheral vascular comorbidities which restricted exercise capacity of the patients. 130 diabetic patients fulfilled our inclusion criteria and were entered the study. These selected patients were requested to undergo standard treadmill exercise test with Bruce protocol.

Before testing, a structured history and medical review were obtained to document symptoms, medical history, medication use, cardiac risk factors, and previous cardiac events and procedures. A questionnaire was designed to record patient’s identification, weight, height, history of hypertension, dyslipidemia, smoking, and chronic kidney disease.

The questionnaire was filled before exercise testing by trained nursing staff of ETT lab. Thereafter patients were instructed to perform 12-lead-ECG exercise tolerance test according to the Bruce protocol. Blood pressure, heart rate, and symptoms were recorded before and during each step of the test, and during recovery period. The 12-lead-ECG was recorded at rest, in each stage of the test, and after the test every minute up to five minute. After test completion, test result was assessed by a trained cardiologist. The same cardiologist analyzed all 130 ETT results. According to ETT interpretation, patients were categorized into negative or positive ETT. The classic criteria for positive stress test were used [J-point and ST80 (defined as the point that is 80ms from the J point) depression of 0.1 mV (1 mm) or more and/or an ST-segment slope within the range of ± 1 mV/s in 3 consecutive beats]. Patients with positive ETT results were advised to undergo coronary angiography.

Angiographic studies were performed in the catheterization laboratory of Taleghani hospital by the attending cardiologist. The procedure was done using Seldinger technique through the femoral artery by Judkins catheters using Iodixanol contrast. Variables in questionnaire and results of ETT and angiographic findings were entered in SPSS statistical software and analyzed.

Finally the positive predictive value of ETT in diabetic patients with atypical symptoms and positive ETT was calculated. The study was approved by the ethics committee of Shaheed Beheshti medical university.

## 4. Results

**Figure 1:**
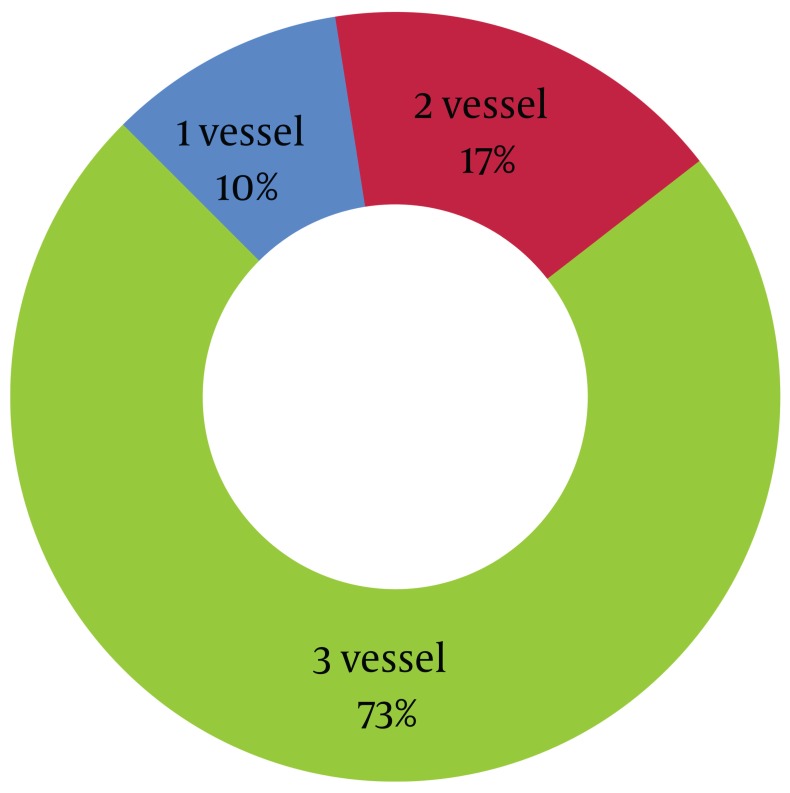
Distribution of 77 Diabetics Patients with Positive ETT and CAD Based on the Number of STenotic Stenosis Vessels Revealed in Angiographic Study.

Among total number of 130 diabetic patients who were entered the study, 100 cases (76.4%) demonstrated positive ETT and underwent coronary angiography. Among diabetic patients with positive ETT, 77% showed definite diagnosis of CAD (defined as involvement of at least one vessel with > 50% reduction in diameter). The positive predictive value of ETT for diagnosis of CAD was 77%. Among the diabetic patients with CAD, according to the angiographic study, 8 patients (10.4%) categorized as single vessel disease, 13 patients (16.9%) as two vessel disease, and 56 patients (72.7%) as 3 vessels stenosis (Figure 1).

There was no significant association between the obesity and observed coronary disease in the diabetic patients. Sex and CAD had no significant correlation (P = 0.3, Table 1). Furthermore, 87% of patients with CAD showed hypertension, and prevalence of hypertension in patients without CAD was 17.4%. (P < 0.001). As shown in Table 1 hypertension was more prevalent in coronary patients compared to those with normal angiogram (OR=31). 97% of patients with CAD and 39.1% of patients with normal angiogram had dyslipidemia. (P < 0.001, OR=58).

**Table 1:** Distribution of Diabetic Patients With or Without CAD in Relation to Their Background Features

Background Features	With CAD (n=77)	Without CAD (n=23)	*P* value	Odds Ratio
**Sex**				
Male	44 (57.1%)	10 (43.5%)	0.3	_
Female	33 (42.9%)	13 (56.5%)		
**HTN**				
Present	67 (87%)	4 (17.4%)	P < 0.000	31
Absent	10 (13%)	19 (82.6%)		
**HLP**				
Present	75 (97.4%)	9 (39.1%)	P < 0.000	58
Absent	2 (2.6%)	14 (60.9%)		
**Obesity**	%3			

## 5. Discussion

Concurrent with advances in the treatment of diabetes, particularly regarding cardiovascular risks, there has been an extraordinary growth in available modalities for diagnosing and treating coronary artery disease (CAD) over the past two decades. This has resulted in significant decline in the mortality caused by CAD during this period of time (13). Unfortunately, for reasons that are not completely understood, this decline has not been appreciated to the same degree by diabetic patients (14).

Despite our efforts to prevent cardiovascular disease, even the most aggressive programs will attenuate but not eliminate the coronary risk. Additionally, many patients with type 2 diabetes have preexisting CAD at the time of diagnosis. Added complexities are diagnostic concerns, such as well recognized atypical, often silent, manifestations of IHD in diabetic patients (15).

Because of the frequency of CAD in patients with diabetes and the potential difficulties involved in its evaluation, the development of accurate and cost effective diagnostic tests is of obvious clinical importance. Options available to evaluate patients non-invasively for CAD have gradually expanded over the time. ETT lacks the specificity to be cost-effective when screening low-risk populations while lacks the sensitivity to be consistently predictive in high-risk groups. As a result, the ETT is most useful in patients with moderate CAD risk (16). The ETT is preferable to a pharmacological stress test because it depicts the heart’s actual workload and better simulates daily cardiac workload (17). Some patients may not be able to exercise on standard treadmill because of underlying peripheral vascular disease (PVD), or pulmonary, neurological, or orthopedic conditions. Others may be unable to reach the target workload required to validate the test results. Still others may have preexisting abnormalities in the resting ECG that obscure any changes induced by exercise. Many of these concerns apply to a diabetic patient who is inherently at high risk for developing CAD and whose exercise potential is often limited by obesity, deconditioning, PVD, or peripheral neuropathy.

A completely normal ETT has been reported to be a marker for a good prognosis in patients with diabetes (18). Patients with diabetes mellitus constitute a group at high risk for CAD and can present a challenge to conventional ETT because of limitations related to comorbidities. However, some data indicate that well-judged patient selection has been associated with safe and accurate evaluation by ETT (19). Many studies have investigated the presence of unknown asymptomatic coronary heart disease (CHD) among diabetic subjects (20-23). In patients with type 2 diabetes mellitus, CAD is generally detected at an advanced stage with extensive atherosclerosis and poor outcomes, whereas CAD is commonly missed in its asymptomatic stages (24). Diabetic patients with asymptomatic CAD have a higher cardiac mortality risk than those with the symptomatic disease (25). Performing routine screening for asymptomatic CAD in all patients with type 2 DM is debatable for several reasons (26-28). CAD screening might identify individuals who could benefit from anti-ischemia therapy or from revascularization, and specifically those with left main or severe multi-vessel disease (28). As mentioned before in this study, we aimed to measure positive predictive value of ETT in diabetic patients. Relationship of other measures such as obesity, sex, hypertension, and dyslipidemia with CAD in diabetic patients was assessed. According to the study results, there is significant association between hypertension and dyslipidemia with CAD in diabetic patients, and presence of these variables significantly increased the risk of CAD in the study population. The same correlation exists in non- diabetic patients with CAD .Sex and obesity had no significant association with CAD in diabetic patients. This result is in contrast with the findings in general population where male sex and obesity are independent risk factors for coronary artery disease and may be partly explainable by overshadowing the presence of diabetes minimizing effects of these risk factors. Hazard of dyslipidemia in developing CAD in diabetic patients was stronger than hypertension. (Odds ratio for dyslipidemia and hypertension were 58 and 31, respectively). So, the study shows the higher risk for development of CAD in diabetic patients with such co morbidities as hypertension and dyslipidemia. In our study positive predictive value of stress testing in diabetic patients was 77%. In a previous study (29) the PPV of ETT in diabetics was 73% which is in accordance with our results. The PPV and NPV of ETT in diabetic and non-diabetic patients were similar. The PPV and NPV in non-diabetic group were 78% and 55%, respectively (30). In the contrary, some other studies demonstrated lower values of PPV in diabetics but it should be mentioned that in most of these studies asymptomatic subjects were tested resulting in a lower PPV compared to symptomatic subjects (31).

The routine screening of asymptomatic patients with type 2 DM and normal ECGs remains controversial and generally are not recommended (32). Due to common occurrence of silent myocardial ischemia in diabetic patients and acceptable PPV of ETT in these patients, screening of CAD with ETT in diabetics seems reasonable in the presence of mild or atypical symptoms, concomitant risk factors, and long lasting diabetes. According to our findings risk factors such as hypertension and dyslipidemia strongly accompany CAD in diabetics; consequently intensive risk factor modification in patients with DM is recommended.

We -acknowledge that our study had some major limitations. First, due to ethical and practical issues we were unable to evaluate NPV of ETT. Second, our sample populations were referred to an outpatient clinic in a teaching hospital which is not a good representative of general population. Third, the patients were not stratified regarding duration and quality of control of diabetes, two important contributors to end organ damage.
